# Association of serum bilirubin levels with risk of cancer development and total death

**DOI:** 10.1038/s41598-021-92442-2

**Published:** 2021-06-24

**Authors:** Toyoshi Inoguchi, Yasunobu Nohara, Chinatsu Nojiri, Naoki Nakashima

**Affiliations:** 1 Fukuoka City Medical Association, Fukuoka City Health Promotion Support Center, Maizuru 2-5-1, Chuou-ku, Fukuoka, 810-0073 Japan; 2 Faculty of Advanced Science and Technology, Kumamoto University, Kumamoto, Japan; 3 Medical Information Center, Kyushu University Hospital, Fukuoka, Japan

**Keywords:** Biomarkers, Oncology, Risk factors

## Abstract

Serum levels of bilirubin, a strong antioxidant, may influence cancer risk. We aimed to assess the association between serum bilirubin levels and cancer risk. Data were retrieved from 10-year electronic medical records at Kyushu University Hospital (Japan) for patients aged 20 to 69 years old. The associations of baseline bilirubin levels with cancer risk (lung, colon, breast, prostate, and cervical) were evaluated using a gradient boosting decision tree (GBDT) model, a machine learning algorithm, and Cox proportional hazard regression model, adjusted for age, smoking, body mass index, and diabetes. The number of study subjects was 29,080. Median follow-up time was 4.7 years. GBDT models illustrated that baseline bilirubin levels were negatively and non-linearly associated with the risk of lung (men), colon, and cervical cancer. In contrast, a U-shaped association was observed for breast and prostate cancer. Cox hazard regression analyses confirmed that baseline bilirubin levels (< 1.2 mg/dL) were negatively associated with lung cancer risk in men (HR = 0.474, 95% CI 0.271–0.828, *P* = 0.009) and cervical cancer risk (HR = 0.365, 95% CI 0.136–0.977, *P* = 0.045). Additionally, low bilirubin levels (< 0.6 mg/dL) were associated with total death (HR = 1.744, 95% CI 1.369–2.222, *P* < 0.001). Serum bilirubin may have a beneficial effect on the risk of some types of cancers.

Bilirubin is the end-product of heme catabolism, and it was considered to have no physiological functions for many years. However, an antioxidant effect of bilirubin was first proposed in 1954^1^. Stocker et al. reported that bilirubin is an effective antioxidant that successfully scavenges peroxyl radicals and suppress the oxidation of lipids and lipoproteins^2^, and the protective effect of bilirubin against oxidative stress-related damages has received increasing attention for the last two decades^3–6^. Various clinical studies have shown that serum bilirubin levels are negatively associated with oxidative stress-related diseases, such cardiovascular diseases, diabetes, diabetic vascular complications, chronic kidney disease, and chronic obstructive pulmonary disease^3–6^.

Although the role of oxidative stress in the pathogenesis of several cancers is well established^7,8^, the relationship between serum bilirubin levels and cancer risk is poorly understood. A 10-year follow-up retrospective population-based cohort study from Belgium showed a significant negative association of serum bilirubin levels with all-cause and cancer mortality in men but not in women^9^. A recent report also showed a strong negative association of serum bilirubin levels with total and cancer mortality in a nested case–control study^10^. In contrast, there has been little evidence regarding the association between serum bilirubin levels and cancer development. Several cohort studies indicated an negative association between baseline serum bilirubin levels and the risk of lung and colon cancer^6,11,12^. However, one prospective study has shown no association between baseline serum bilirubin levels and colon cancer risk in the National Health and Nutritional Examination Survey cohort^13^, and another case-cohort study showed that serum bilirubin levels were not associated with any cancer risk (breast, prostate, colon, or lung) or cancer mortality^14^. In recent years, several studies have investigated the potential causal associations of bilirubin levels with the risk of lung and colon cancer by examining whether genetically raised serum bilirubin levels affect cancer risk in a Mendelian randomization analysis^15–17^. However, the results remained still inconclusive.

Recently, a machine learning approach has been increasingly used in medical fields. The conventional statistical method for making predictions involves generalized linear models. However, the relationship between explaining variables and outcome variable is largely non-linear in clinical setting. The machine learning technique is relatively free of this limitation of statistical analysis. If the relationship between serum bilirubin levels and risk of cancer development is non-linear, the machine learning approach may be useful for evaluation of such relationship.

In this study, we therefore examined the predictive value of baseline bilirubin levels for the development of various types of cancer (lung, colon, breast, prostate, and cervical) and total death in a hospital-based data-driven large-scale cohort study using both a gradient boosting decision tree (GBDT) model, which is a machine learning algorithm^18^, and conventional Cox proportional hazard regression models.

## Results

### Characteristics of study subjects

A total of 29,080 subjects (12,946 men and 16,134 women) were eligible for inclusion in the analysis (Fig. 1S in the Supplements). Table 1 shows the baseline characteristics of the study subjects. The median age was 52 years old, and the median follow-up time was 4.7 years. There were 403 lung (247 men, 156 women), 315 colon (173 men, 142 women), 275 breast (9 men, 266 women), 269 prostate, and 104 cervical cancer cases and 331 total deaths (212 men, 119 women) in this study.

**Table 1:** Baseline characteristics of the study subjects.

	Total	Men	Women
No	29,080	12,946	16,134
Follow-up time, years	4.7 (2.4–7.9)	4.6 (2.3–7.7)	4.8 (2.5–8.0)
Age, years	52 (37–62)	55 (42–63)	49 (34–60)
Body mass index, kg/m^2^	23.0 (4.2) (*n* = 28,105)	23.7 (3.8) (*n* = 12,532)	22.4 (4.3) (*n* = 15,573)
Bilirubin, mg/dL	0.65 (0.50–0.82)	0.70 (0.54–0.90)	0.60 (0.50–0.80)
Smoking, yes	8301(31.8%) (*n* = 26,144)	6021 (52.0%) (*n* = 11,576)	2280 (15.7%) (*n* = 14,568)
Diabetes, yes	5120 (17.6%)	2958 (22.8%)	2162 (13.4%)
**Incidence case No**			
Lung cancer	403	247	156
Colon cancer	315	173	142
Breast cancer	275	9	266
Prostate cancer	269	269	–
Cervical cancer	104	–	104
Total all-cause death	331	212	119

Data was expressed as median (IQR) or mean (SD). The number (No.) was expressed as absolute value and %.

### SHAP summary plots of cancer risk in GBDT models

**Figure 1:**
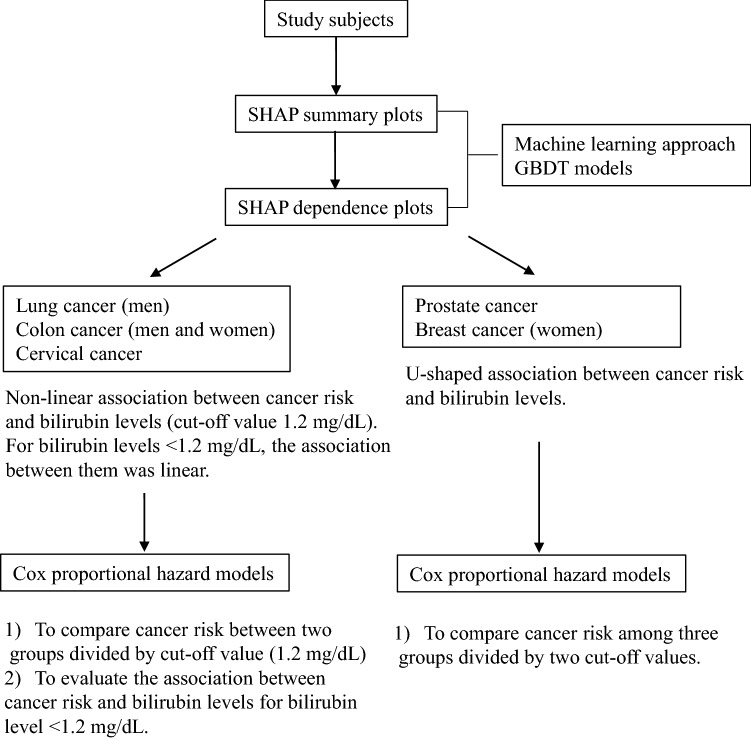
The roadmap for statistical analyses to evaluate the association between each cancer risk and serum bilirubin levels. GBDT model, gradient boosting decision tree model; SHAP summary plots, SHapley Additive exPlanations summary plots; SHAP dependence plots, SHapley Additive exPlanations summary plots.

**Figure 2:**
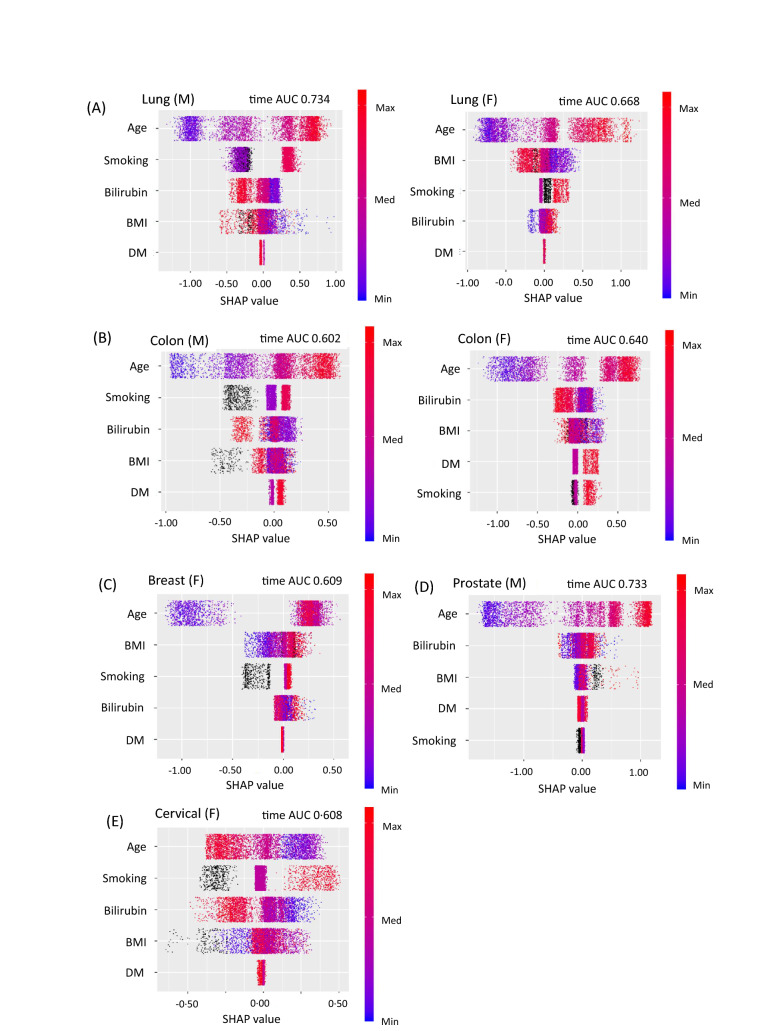

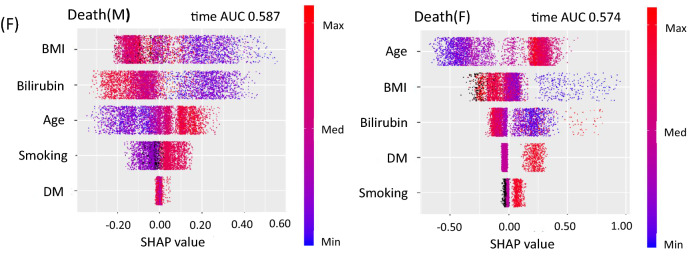
SHAP summary plots for cancer risk. SHAP values are shown on the x-axis and represent the contribution weight to each cancer risk in descending order. Red dot, the maximum value of a variable; blue dot, the minimum value of a variable; black dot, an unknown value of a variable. (**A**) lung cancer in men (M) and women (F), (**B**) colon cancer in men (M) and women (F), (**C**) breast cancer, (**D**) prostate cancer. (**E**) cervical cancer, (**F**) total all-cause death in men (M) and women (F). SHAP value, SHapley Additive exPlanations; Bilirubin, serum bilirubin levels; BMI, body mass index; DM, diabetes; AUC, area under the curve.

First, we generated a prediction model for each cancer risk using GBDTs (Fig. 1). Figure 2 shows SHapley Additive exPlanation (SHAP) summary plots for each variable. This is a recent method of interpreting the outcome of a machine learning model^19,20^. The SHAP summary plots represents the contribution weight to each cancer risk in descending order, and visually illustrated the associations between each variable and cancer risk. Specifically, as shown in Fig. 2A, lung cancer risk in men appeared to be apparently associated with low bilirubin levels in addition to age and smoking, but not in women. Colon cancer risk appeared to be associated with low bilirubin levels in addition to age in women, and similar association was observed in men in addition to age and smoking (Fig. 2B). Cervical cancer risk also appeared to be associated with low bilirubin levels in addition to age and smoking (Fig. 2E). In contrast, breast cancer appeared to be associated with only age and BMI (Fig. 2C), and prostate cancer risk appeared to be associated with only age, but not bilirubin levels, as shown in Fig. 2D.

### SHAP dependence plots of cancer risk in GBDT models

**Figure 3:**
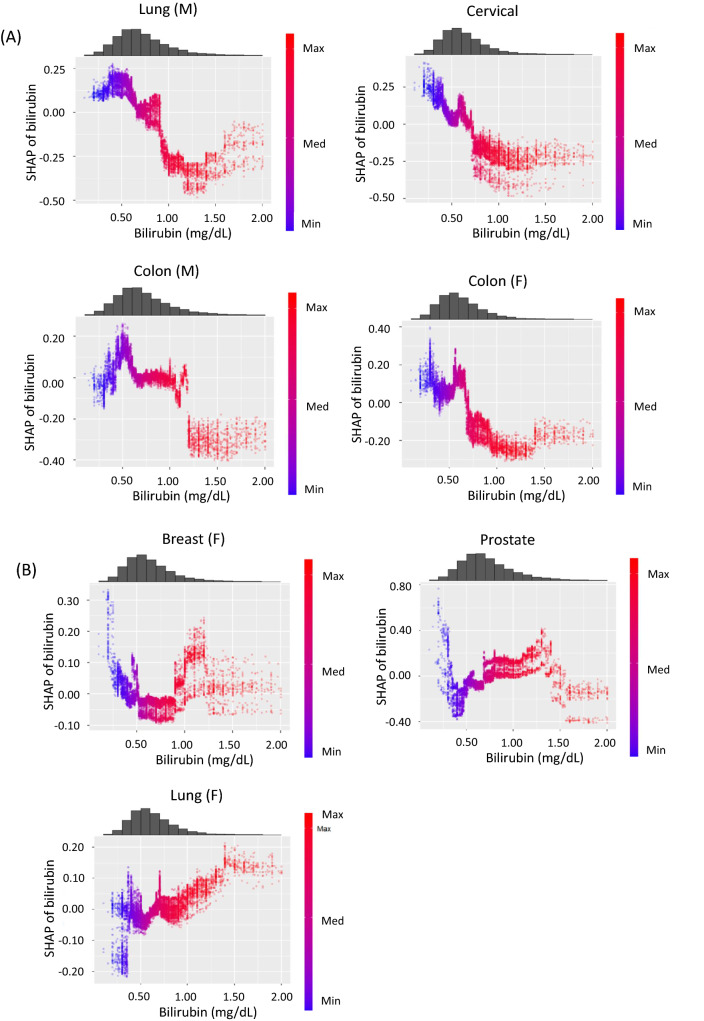

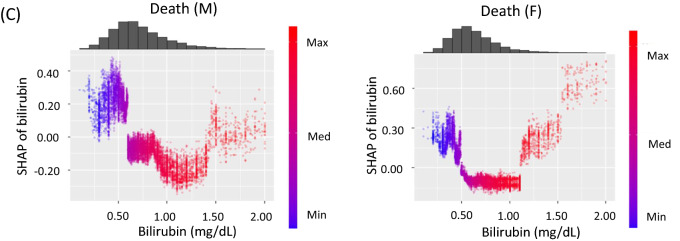
SHAP dependence plots for cancer risk against serum bilirubin levels. SHAP values are shown on the y-axis. (**A**) lung cancer in men (M), cervical cancer, colon cancer in men (M), and colon cancer in women (F). (**B**) breast cancer in women (F), prostate cancer, and lung cancer in women (F). (**C**) total all-cause death in men (M) and women (F). SHAP, SHapley Additive exPlanations.

To focus on the association between serum bilirubin levels and each cancer risk, SHAP dependence plot (SDP) analyses were performed^21^. Of interest, the associations were clearly classified into two patterns. First, bilirubin levels were negatively and non-linearly associated with a risk of lung cancer in men, colon cancer in men and women, and cervical cancer (Fig. 3A). These negative associations were approximately linear for bilirubin levels < 1.2 mg/dL, and the lowest risk was observed in patients with high bilirubin levels ≥ 1.2 mg/dL. In contrast, these analyses clearly revealed that there was a U-shaped association for prostate cancer and breast cancer risk (Fig. 3B) and there was no association between bilirubin levels and lung cancer risk in women (Fig. 3B).

### Cox hazard regression analysis

The SDP analyses in GBD models showed the non- linear relationship between serum bilirubin levels and cancer risks. First, according to the SDP analyses, the association patterns between bilirubin levels and cancer risk were divided into two patterns by the cut-off value (1.2 mg/dL) for lung cancer in men, colon cancer, and cervical cancer, and Cox hazard regression analyses were conducted (Fig. 1). Subjects with high bilirubin levels > 1.2 mg/dL were associated with lung cancer risk in men [hazard ratio (HR) = 0.434, 95% CI 0.214–0.880, *P* < 0.001] and colon cancer risk (HR = 0.429, 95% CI 0.212–0.868, *P* = 0.019), and tended to be associated with cervical cancer risk (HR = 0.285, 95% CI 0.040–2.049, *P* = 0.213) (Table 2). For bilirubin levels < 1.2 mg/dL, the analyses were performed using serum bilirubin levels as continuous variables because the associations were approximately linear. As a result, serum bilirubin levels were negatively associated with lung cancer risk in men (HR = 0.474, 95% CI 0.271–0.828, *P* = 0.009) and cervical cancer risk (HR = 0.365, 95% CI 0.136–0.977, *P* = 0.045) and tended to be associated with colon cancer risk (HR = 0.647, 95% CI 0.391–1.070, *P* = 0.090) (Table 2).

**Table 2:** The associations between serum bilirubin levels and each cancer risk (lung in men, colon, and Cervical).

Variables	HR	95% CI		*P*	Variables	HR	95% CI		*P*
Comparison in cancer risk between two BIL groups (BIL ≥ 1.2 and < 1.2) divided by cut-off value					Association between BIL and cancer risk (BIL < 1.2 mg/dL)				
**Lung cancer (M)**					**Lung cancer (M)**				
Age (20–39)	0.288	0.143	0.579	< 0.001	Age (20–39)	0.283	0.140	0.573	< 0.001
Age (60–69)	2.504	1.874	3.348	< 0.001	Age (60–69)	2.566	1.918	3.432	< 0.001
BMI (< 21)	1.316	0.976	1.775	0.071	BMI (< 21)	1.278	0.947	1.725	0.109
BMI (≥ 27)	0.752	0.482	1.171	0.207	BMI (≥ 27)	0.761	0.488	1.186	0.227
BIL (≥1.2)	0.434	0.214	0.880	0.021	BIL	0.474	0.271	0.828	0.009
Smoking	2.883	2.118	3.925	< 0.001	Smoking	2.846	2.090	3.876	< 0.001
Diabetes	0.834	0.618	1.125	0.235	Diabetes	0.819	0.606	1.106	0.193
**Colon cancer**					**Colon cancer**				
Age (20–39)	0.348	0.227	0.534	< 0.001	Age (20–39)	0.345	0.225	0.529	< 0.001
Age (60–69)	1.481	1.161	1.890	0.002	Age (60–69)	1.504	1.178	1.920	0.001
BMI (< 21)	1.203	0.928	1.558	0.162	BMI (< 21)	1.188	0.917	1.538	0.192
BMI (≥ 27)	0.860	0.595	1.245	0.424	BMI (≥ 27)	0.857	0.592	1.240	0.413
BIL (≥ 1.2)	0.429	0.212	0.868	0.019	BIL	0.647	0.391	1.070	0.090
Smoking	1.469	1.137	1.897	0.003	Smoking	1.460	1.130	1.887	0.004
Diabetes	1.456	1.129	1.878	0.004	Diabetes	1.445	1.119	1.866	0.005
Sex	1.310	1.008	1.704	0.044	Sex	1.322	1.014	1.722	0.039
**Cervical cancer**					**Cervical cancer**				
Age (20–39)	1.707	1.102	2.646	0.017	Age (20–39)	1.644	1.059	2.551	0.027
Age (60–69)	0.593	0.319	1.102	0.098	Age (60–69)	0.616	0.331	1.147	0.127
BMI (< 21)	0.729	0.473	1.123	0.151	BMI (< 21)	0.730	0.474	1.124	0.153
BMI (≥ 27)	0.918	0.494	1.707	0.787	BMI (≥ 27)	0.895	0.481	1.665	0.726
BIL (≥ 1.2)	0.285	0.040	2.049	0.213	BIL	0.365	0.136	0.977	0.045
Smoking	2.375	1.540	3.662	< 0.001	Smoking	2.298	1.487	3.551	< 0.001
Diabetes	0.932	0.521	1.669	0.813	Diabetes	0.907	0.507	1.625	0.744

For lung cancer in men (M), colon cancer, and cervical cancer, the association between and cancer risk was evaluated by Cox hazard regression models. (Left table) First, serum bilirubin levels were divided into two groups using the cut-off value (1.2 mg/dL). The reference group for serum bilirubin levels was that of < 1.2 mg/dL. BIL (≥ 1.2), subjects with bilirubin levels ≥ 1.2 mg/dL. (Right table) Next, for serum bilirubin levels < 1.2 mg/dl, they were used as continuous variables (BIL), and the association between bilirubin levels and each cancer risk was evaluated. Age (20–39), subjects aged 20–39 years old; Age (60–69), subjects aged 60–69 years old; BMI (< 21), subjects with body mass index < 21 kg/m^2^; BMI (≥ 27), subjects with body mass index ≥ 27 kg/m^2^. The reference of age and BMI was a middle range group.

In contrast, since a U-shaped association was observed for prostate cancer and breast cancer, serum bilirubin levels were divided into three groups by the cut-off values in each cancer separately prior to analysis. Prostate cancer risk was associated with subjects with high bilirubin levels ≥ 0.7 mg/dL (HR = 1.348, 95% CI 1.021–1.780, *P* = 0.035). Additionally, subjects with low bilirubin levels < 0.4 mg/dL tended to be associated with prostate cancer risk (HR = 1.407, 95% CI 0.795–2.489, *P* = 0.241). Breast cancer risk also tended to be associated with high bilirubin levels (≥ 0.9 mg/dL) (HR = 1.306, 95% CI 0.913–1.867, *P* = 0.144). No significant association was found between serum bilirubin levels and lung cancer risk in women (Table 3).

**Table 3:** The associations between serum bilirubin levels and each cancer risk (prostate, breast, and lung cancer in women).

Variables	HR	95% CI		*P*
Comparison in cancer risk among three BIL groups (low, middle, and high) divided by two cut-off values				
**Prostate cancer**				
Age (20–39)	0.088	0.028	0.281	< 0.001
Age (60–69)	2.684	2.011	3.583	< 0.001
BMI (< 21)	0.945	0.680	1.312	0.733
BMI (≥ 27)	0.765	0.504	1.162	0.209
BIL (< 0.4)	1.407	0.795	2.489	0.241
BIL(≥ 0.7)	1.348	1.021	1.780	0.035
Smoking	0.927	0.716	1.200	0.565
Diabetes	0.908	0.677	1.219	0.522
**Breast cancer**				
Age (20–39)	0.458	0.319	0.658	< 0.001
Age (60–69)	1.181	0.891	1.565	0.248
BMI (< 21)	0.748	0.567	0.988	0.041
BMI (≥ 27)	0.919	0.615	1.373	0.679
BIL (< 0.6)	1.122	0.846	1.489	0.424
BIL (≥ 0.9)	1.306	0.913	1.867	0.144
Smoking	1.180	0.837	1.662	0.345
Diabetes	0.837	0.585	1.198	0.331
**Lung cancer (F)**				
Age (20–39)	0.261	0.135	0.504	< 0.001
Age (60–69)	2.413	1.678	3.469	< 0.001
BMI (< 21)	1.303	0.919	1.848	0.137
BMI (≥ 27)	0.588	0.291	1.188	0.139
BIL (< 0.5)	1.029	0.680	1.556	0.893
BIL (≥ 0.9)	1.041	0.645	1.680	0.871
Smoking	2.004	1.340	2.997	0.001
Diabetes	0.885	0.560	1.398	0.600

For prostate cancer, breast cancer, and lung cancer in women, serum bilirubin levels were divided into three groups by the two cut-off values. BIL (< 0.4, 0.5, or 0.6), subjects with bilirubin levels < 0.4, 0.5, or 0.6 mg/dL; BIL (≥ 0.7 or 0.9); subjects with bilirubin levels ≥ 0.7, or 0.9. The reference group for serum bilirubin levels was a middle range group. Age (20–39), subjects aged 20–39 years old; Age (60–69), subjects aged 60–69 years old; BMI (< 21), subjects with body mass index < 21 kg/m^2^; BMI (≥ 27), subjects with body mass index ≥ 27 kg/m^2^. The reference of age and BMI was a middle range group.

### Association between serum bilirubin levels and total death

The SHAP summary plots showed that total death appeared to be associated with low BMI, low bilirubin levels, age, and smoking in descending order in men, and it appeared to be associated with age, low BMI, low bilirubin levels, diabetes, and smoking in women (Fig. 2F). The SDP analysis clearly showed that there was a reverse J-shaped association for total death in men and a U- shaped association in women (Fig. 3C). Thus, bilirubin levels were divided into three groups using the two cut-off values (0.6 and 1.1 mg/dL). Cox hazard regression analyses showed that subjects with low bilirubin levels < 0.6 mg/dL were associated with total death risk in men (HR = 1.998, 95% CI 1.481–2.694, *P* < 0.001), whereas subjects with high bilirubin levels ≥ 1.1 mg/dL tended to be negatively associated with total death risk (HR = 0.623, 95% CI 0.332–1.170, *P* = 0.141). In women, subjects with high bilirubin levels ≥ 1.1 mg/dL were associated with total death (HR = 2.448, 95% CI 1.286–4.661, *P* = 0.006), and subjects with low bilirubin levels tended to also be associated with its risk (HR 1.371, 95% CI 0.909–2.067, *P* = 0.132). In a combined analysis of men and women, subjects with low bilirubin levels < 0.6 mg/dL were associated with total death (HR = 1.744, 95% CI 1.369–2.222, *P* < 0.001) (Table 4).

**Table 4:** Association between serum bilirubin levels and total all-cause death in men, women, and all subjects.

Variables	HR	95% CI		*P*
Comparison in cancer risk among three BIL groups (low, middle, and high) divided by two cut-off values				
**Total death (M)**				
Age (20–39)	0.748	0.488	1.148	0.185
Age (60–69)	1.302	0.951	1.783	0.100
BMI (< 21)	1.716	1.252	2.353	0.001
BMI (≥ 27)	1.085	0.696	1.692	0.718
BIL (< 0.6)	1.998	1.481	2.694	< 0.001
BIL (≥ 1.1)	0.623	0.332	1.170	0.141
Smoking	1.248	0.931	1.672	0.138
Diabetes	1.013	0.731	1.405	0.938
**Total death (F)**				
Age (20–39)	0.461	0.271	0.784	0.004
Age (60–69)	1.029	0.671	1.575	0.897
BMI (< 21)	1.469	0.974	2.216	0.067
BMI (≥ 27)	1.090	0.591	2.009	0.782
BIL (< 0.6)	1.371	0.909	2.067	0.132
BIL (≥ 1.1)	2.448	1.286	4.661	0.006
Smoking	1.263	0.778	2.049	0.345
Diabetes	2.217	1.446	3.398	< 0.001
**Total death (T)**				
Age (20–39)	0.604	0.433	0.844	0.003
Age (60–69)	1.197	0.931	1.540	0.161
BMI (< 21)	1.627	1.265	2.093	< 0.001
BMI (≥ 27)	1.079	0.753	1.544	0.679
BIL (< 0.6)	1.744	1.369	2.222	< 0.001
BIL (≥ 1.1)	1.010	0.646	1.579	0.964
Smoking	1.247	0.971	1.600	0.083
Diabetes	1.317	1.015	1.708	0.038
Sex, men	2.200	1.689	2.866	< 0.001

The associations between serum bilirubin levels and total all-cause death in men (M), women (F), and all patients (T). Serum bilirubin levels were divided into three groups by the two cut-off values of 0.6 mg/dL and 1.1 mg/dL. The reference group for serum bilirubin levels was a middle range group. Age (20–39), subjects aged 20–39 years old; Age (60–69), subjects aged 60–69 years old; BMI (< 21), subjects with body mass index < 21 kg/m^2^; BMI (≥ 27), subjects with body mass index > 27 kg/m^2^; BIL (< 0.6), subjects with bilirubin levels < 0.6 mg/dL; BIL (≥ 1.1); subjects with bilirubin levels > 1.1. The reference of age and BMI was a middle range group.

## Discussion

In this study, we showed the non-linear relationship between serum bilirubin levels and cancer risks using used SHAP summary plots and SDPs calculated from the GBDT model as estimation methods, which have been recently developed to improve the interpretability of outputs from machine learning approach and its consistency with human intuition^19–21^. These analyses enabled us to use linear regression models appropriately. Thus, Cox proportional hazard regression models showed that bilirubin levels were negatively associated with an increased risk of lung cancer in men and cervical cancer in the range of bilirubin levels < 1.2 mg/dL. A similar association was observed in colon cancer risk, although it was not statistically significant (HR = 0.647, *P* = 0.090). In addition, subjects with high bilirubin levels ≥ 1.2 mg/dL was significantly associated with the decreased risk for lung cancer in men and colon cancer. Subjects with high bilirubin levels ≥ 1.2 mg/dL were thought to be those with Gilbert’s syndrome, a congenital mild hyperbilirubinemia^22,23^, because subjects with abnormally high bilirubin levels who had diseases, including liver cirrhosis, hemolytic anemia, or other hepatobiliary diseases, were excluded in this study. Subjects with Gilbert’s syndrome may have a lower risk of lung cancer in men and colon cancer.

Cancer-associated infections, smoking, obesity, diabetes, ionizing and ultraviolet radiation, and air pollution are established risk factors for cancer development^24^. All of these factors are likely to be associated with increased reactive oxygen species (ROS) production in humans. Increased ROS production has been hypothesized to damage DNA, proteins, and lipids, and thus initiate or promote cancer development^7,8^. Since bilirubin is a strong endogenous antioxidant, lower serum bilirubin levels reduce the systemic antioxidant capacity, resulting in an impaired defending ability against oxidative stress-induced damage. Therefore, it is very likely that the associations between lower serum bilirubin levels and increased cancer risk are mediated by decreased antioxidant activities.

Serum bilirubin levels are influenced by many environmental factors, including physiological and pathological conditions, as well as genetic factors. It has been reported that smoking is negatively associated with bilirubin levels^25,26^. In addition, low bilirubin levels have been reported in patients with various chronic diseases and conditions, such as diabetes, obesity, aging-related disability^3–6,27^. Therefore, the association between low bilirubin levels and cancer risk may be mediated, at least in part, by the effect of these factors. However, the present study revealed that the association remained significant even after the model was adjusted for these variables. Taken together, low bilirubin levels may reflect a total susceptibility determined by both genetics and various environmental factors to some types of cancers, and thus might be a clinically useful biomarker for the risk of cancer development.

In contrast, a U-shaped association was observed for breast cancer and prostate cancer in the GBDT model. The increased risk of these cancers in patients with high bilirubin levels was inconsistent with the concept of the protective effect of bilirubin. Of great interest, breast cancer and prostate cancer are both estrogen-dependent cancers. Estrogens are well-known risk factors for breast cancer^28^, and previous epidemiologic and experimental findings have indicated key roles of estrogens in prostate cancer development and progression^29,30^. Of note, both serum bilirubin levels and estrogen activities are mainly regulated by uridine diphosphate-glucuronosyltransferase 1A1 (UGT1A1). Serum bilirubin levels are highly related to genetics, and many genome-wide association studies have shown the substantial contribution of various UGT1A1 polymorphisms to human serum bilirubin levels^31^. Serum estrogen activities are also regulated by UGT1A1 by the conjugation and subsequent direct inactivation of estrogens^32^. Therefore, it is very likely that high bilirubin levels due to UGT1A1 polymorphisms may be accompanied by high activities of serum estrogens and a subsequently increased risk of estrogen-dependent cancers. In fact, several studies have shown that increased estrogen activities due to UGT1A1 polymorphisms may be associated with an increased risk of breast cancer, although this phenomenon is still controversial^33,34^. Recent study investigated the relationship between genetically raised bilirubin levels and risk of 10 cancer. This study showed that genetically raised bilirubin levels were negatively associated with squamous cell lung cancer risk and positively associated with breast cancer risk, but not associated with prostate cancer risk^35^. To our knowledge, our study is the first to show the association between high serum bilirubin levels and prostate cancer risk. As for lung cancer in women, non-smoking lung adenocarcinoma was reported to be strongly associated with the female sex, and estrogens are suggested to be involved in the development of this type of lung cancer in women^36,37^. This might offset the association between low bilirubin levels and lung cancer risk observed in men. However, these hypotheses should be confirmed in further studies.

In this study, we also showed that subjects with serum bilirubin levels < 0.6 mg/dL were associated with an increased risk of total all-cause death in men and total subjects, and a U-shaped association was observed in women. Since cancer is a significant cause of death (~ 27%) (https://www.mhlw.go.jp/toukei/saikin/hw/jinkou/geppo/nengai18) in Japan, these associations might be primarily explained by cancer mortality. However, since low bilirubin levels are associated with cardiovascular diseases and other chronic diseases, the detailed cause of death related to bilirubin levels should be evaluated in future studies.

The present study had some merits. First, a key strength of this study is that statistical evaluations were performed using a combination of machine learning and classical statistical approaches. The associations of low serum bilirubin levels with some types of cancer risk found in this study were quite different from the previous some studies showing that bilirubin levels were not associated with any cancer risk including colon, lung, breast or prostate^13,14,35^. This difference may be due to no consideration to the non-linear association in that study. The similarities and differences between this study and previous studies were summarized in Supplement Table 1S. Second, the important merit of this study was its setting in practical care. Regular clinical visits and hospitalizations could lead to a higher chance of an early and accurate diagnosis of cancer development and thus could reduce the misdiagnosis of indolent cancers. Third, the longitudinal study design and exclusion of cancer cases diagnosed within 1 year from enrollment minimized the potential reverse causality. There are several limitations to this study. First, we used EMR data from one hospital. Studies using EMR have the potential for confounding bias due to a lack of randomization and for selection bias, and there were missing data on many parameters. Second, the study was not prospective. Third, the sample size was not large enough to evaluate non-linear and complicated associations between bilirubin levels and some cancer risks using a classic statistical approach. Forth, the study subjects were suffering from different kinds of chronic diseases. Whether the results of this study can be generalized to healthy subjects without any chronic diseases remains to be elucidated.

In conclusion, combination approach using both machine learning methods and conventional statistical analysis showed that baseline serum bilirubin levels were negatively and non-linearly associated with some types of cancer risk including lung cancer in men, cervical cancer, and probably colon cancer. The causal association between serum bilirubin levels and cancer risk and its clinical utility should be evaluated in future prospective studies.

## Methods

### Study subjects

We obtained data from the electronic medical record (EMR) system at Kyushu University Hospital (Japan) for 311,391 patients between January 1st, 2008 and December 31th, 2017. This practical care information included age, sex, height, weight, smoking status, diagnoses [International Classification of Disease version 10 (ICD-10) codes], laboratory test results, and details of prescription medications. Eligible patients were 20 to 69 years old (*n* = 203,104) and had recorded serum bilirubin levels (*n* = 10,8014). In addition, the patients, who had a history of admission and were followed up for over 1 year, were included in the analysis to increase the accuracy of their information (*n* = 41,415) (Fig. 1S in the Supplement). Patients were excluded if they had a previous history of cancers or had ICD-10 codes corresponding to liver cirrhosis or hemolytic anemia or had other hepatobiliary diseases with abnormal liver enzyme levels (alanine aminotransferase or alkaline phosphatase greater than twofold of the upper limit of the normal range). Cancer cases diagnosed within 1 year from recruitment into the study were excluded to minimize potential reverse causality. In addition, patients with serum bilirubin levels over 2.0 mg/dL were excluded because those patients may have had unidentified pathological conditions affecting serum bilirubin levels, although some of them had hereditary hyperbilirubinemia, such as Gilbert’s syndrome (Fig. 1S in the Supplement). All procedures were performed in accordance with the relevant guidelines and regulations. Informed consent was observed from all participants and/or their legal guardians. The study was approved by the ethics committee of Kyushu University Hospital.

### Procedure

The outcomes of this study were the new development of cancers (lung, colon, breast, prostate, and cervical) and all-cause death. To identify incident cancer cases, we obtained the ICD-10 code of each cancer and death from the EMR. For colon cancer, patients with familial adenomatous polyposis were excluded. Follow-up times for each patient were calculated as the time between study enrollment and the date of either outcome onset or the last contact. For baseline serum bilirubin levels of each patient, we used the mean value of those tested during the 6 months before enrollment.

### Statistical analysis

Since a non-linear relationship was expected to exist between bilirubin serum levels and cancer risk or total death, we used a GBDT model, one of the tree-based machine learning algorithms for making prediction models^18^. First, we generated a prediction model for the risk of each cancer and total death using extreme gradient boosting (XGBoost) ver. 1.0.0.2 (https://github.com/dmlc/xgboost)^38^, and variables, including serum bilirubin levels, age, BMI, smoking status (current or past), and the presence of diabetes. In the setup of the hyperparameters, the main fine-tuned parameters in this study included learning rate (learning_rate = 0.1) and maximum tree depth (max_depth = 3). All the other parameters remained at their default values of XGBoost. In this study, we implemented SHapley Additive exPlanation (SHAP), which is a recent method of interpreting the outcome of a machine learning model. The SHAP value represents the contribution weight of each variable to the prediction model^19,20^. It is generally considered to be comparable to a standardized partial regression coefficient in linear regression models. Then, to illustrate visually the relationship between bilirubin levels and each cancer risk or total death, we used a SHAP dependence plot (SDP)^21^. In this study, to confirm the results obtained from GBDTs, conventional statistical analyses were performed using Cox hazard regression models adjusted for age, BMI, smoking status, and the presence of diabetes. Since these associations were not linear, cancer risk was compared between two groups determined by the cut-off value of bilirubin levels or three groups determined by the two cutoff values according to the SDP patterns. The roadmap for statistical analyses was shown in Fig. 1. In all analyses, BMI was divided into three groups: low BMI < 21 kg/m^2^, 21 ≤ middle BMI < 27 kg/m^2^, and high BMI ≥ 27 kg/m^2^ according to a previous report showing the relationship between BMI and cancer risk in a Japanese population^39^. Age was divided into three groups: 20 to 39 years old, 40 to 59 years old, and 60 to 69 years old. All statistical analyses were performed using R software ver. 3.6.3 (R Project for Statistical Computing, https://cran.ism.ac.jp/). We considered 2-sided *P* values of less than 0.05 as statistically significant.

## Supplementary Information

Supplementary Information.

## Data Availability

The datasets generated and/or analyzed during the current study are available from the corresponding author on reasonable request.
