# Supplementary material for: Association of serum bilirubin levels with risk of cancer development and total death
Source: Sci Rep. 2021 Jun 24;11:13224. doi: 10.1038/s41598-021-92442-2 (PMC8225648; doi:10.1038/s41598-021-92442-2)
Supplement: Supplementary file 1 — Supplementary Information. [file 41598_2021_92442_MOESM1_ESM.pdf]

Supporting Information

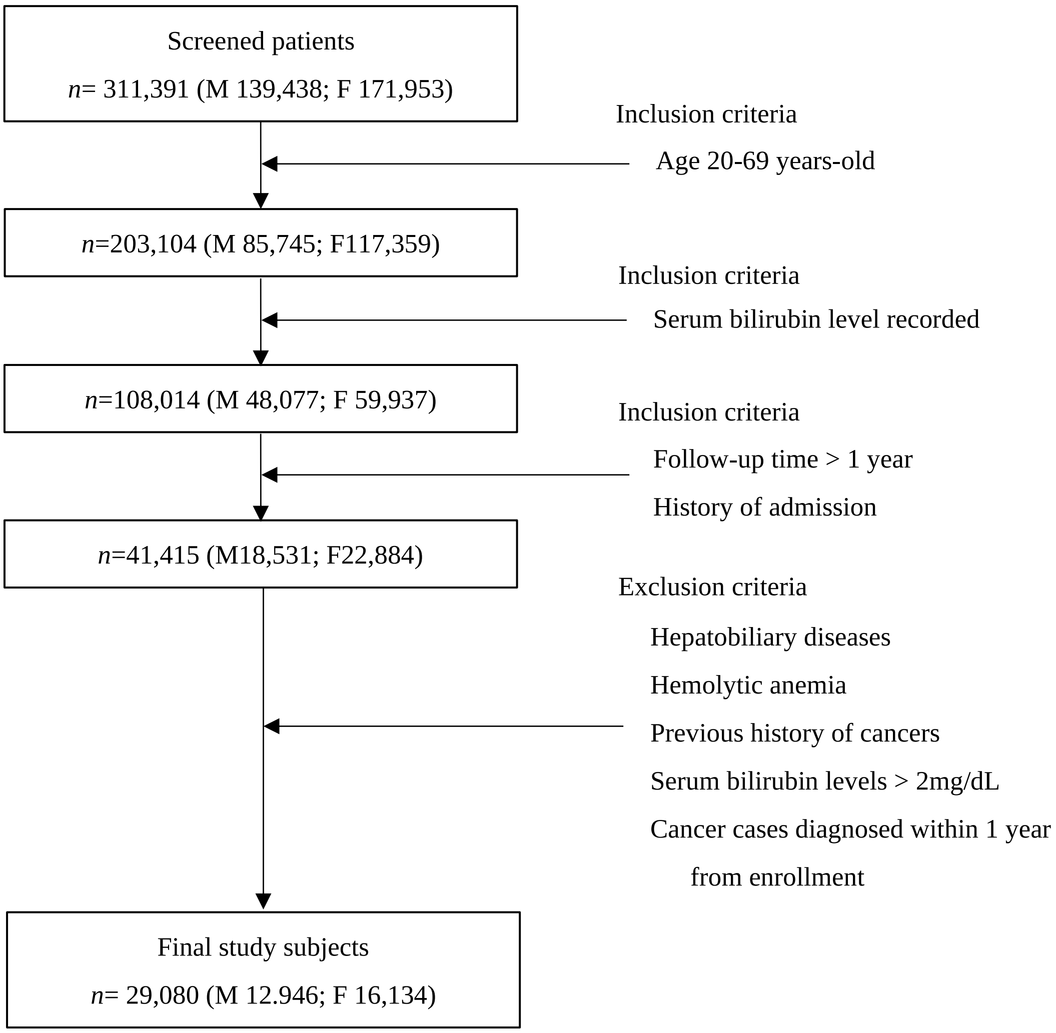

Fig. S1 Selection flow of study subjects

Table 1S Summary of the results regarding the association between cancer risk and serum bilirubin levels in the present study and previous studies.

Cancer type	The present study	Previous studies
Lung cancer	Men<br>・Non-linear association (cut-off value 1.2 mg/dL) between cancer risk and bilirubin levels.<br>・Subjects with bilirubin levels≥1.2 mg/dL was associated with decreased cancer risk.<br>・For bilirubin levels <1.2 mg/dL, bilirubin levels were negatively associated with cancer risk.<br>Women<br>・No association with cancer risk.	・Bilirubin levels were negatively associated with cancer risk (ref 6).<br>・Bilirubin levels were negatively associated with cancer risk in male smoker (ref 12).<br>・Genetically raised bilirubin levels were associated with cancer risk in smokers (ref 15)<br>・No association between cancer risk and bilirubin levels (ref 14).
Colon cancer	・Non-linear association (cut-off value 1.2 mg/dL) between cancer risk and bilirubin levels.<br>・Subjects with bilirubin levels≥1.2 mg/dL was associated with decreased cancer risk.<br>・For bilirubin levels <1.2 mg/dL, bilirubin levels tended to be associated with cancer risk (HR=0.647, *P*=0.090), but it was not statistically significant.	・Bilirubin levels were negatively associated with cancer risk (ref 11).<br>・Genetically raised bilirubin levels were negatively associated with cancer risk in men, but not in women (ref 16).<br>・No association between cancer risk and bilirubin levels (ref 13, 14).
Breast cancer	・U-shaped association between cancer risk and bilirubin levels.<br>・No significant association between cancer risk and bilirubin levels.	・No association between cancer risk and bilirubin levels (ref 14).<br>・Genetically raised bilirubin levels were positively associated with cancer risk (ref 35).
Prostate cancer	・U-shaped association between cancer risk and bilirubin levels.<br>・Subjects with bilirubin levels≥0.7 mg/dL was associated with increased cancer risk.	・No association between cancer risk and bilirubin levels (ref 14, and 35).
Cervical cancer	・Non-linear association (cut-off value 1.2 mg/dL) with cancer risk and bilirubin levels.<br>・For bilirubin levels <1.2 mg/dL, bilirubin levels were negatively associated with cancer risk.	・No report regarding the association between cancer risk and bilirubin levels.

Ref, reference number in the manuscript.
